# The value of preoperative neutrophil/lymphocyte ratio in predicting the severity of cholecystolithiasis with cholecystitis in elderly patients

**DOI:** 10.1186/s12893-023-02267-1

**Published:** 2023-11-27

**Authors:** Zeliang Xia, Yanyu Liu, Siyu Sun, Erbo Shan, Yanhao Liu

**Affiliations:** 1 Department of General Surgery, The Second Affiliated Hospital of Bengbu Medical College, Anhui, Bengbu, 233004 PR China; 2 Department of Intensive Care Unit, The First Affiliated Hospital of Bengbu Medical College, Anhui, Bengbu, 233004 PR China; 3 Department of Pharmacy, The Second Affiliated Hospital of Bengbu Medical College, Anhui, Bengbu, 233004 PR China; 4 Department of Clinical Trial Research Center, The First Affiliated Hospital of Bengbu Medical College, Anhui, Bengbu, 233004 PR China

**Keywords:** Neutrophil-to-lymphocyte ratio, C-reactive protein, Severity of cholecystolithiasis with cholecystitis, Inflammatory biomarkers, Diagnosis

## Abstract

**Background:**

This study aims to assess the effectiveness of neutrophil/lymphocyte ratio (NLR) and C-reactive protein (CRP) in diagnosing cholecystolithiasis with cholecystitis in elderly patients. Additionally, the study seeks to determine the predictive value of preoperative NLR in determining the severity of the condition in this population.

**Methods:**

This study is a retrospective cohort study, including 160 elderly patients with cholecystolithiasis with cholecystitis (45 cases of simple cholecystitis, 58 cases of suppurative cholecystitis, 57 cases of gangrenous cholecystitis) and 60 cases of normal gallbladder histology. The study collected clinical data of the patients detected the preoperative CRP content, neutrophil, and lymphocyte levels through blood routine tests, and calculated the NLR value. The diagnostic value of NLR and CRP was determined by using the Receiver Operating Characteristic Curve (ROC), and the optimal value of preoperative NLR related to the severity of elderly patients with cholecystolithiasis with cholecystitis was identified.

**Results:**

This study found that for elderly patients with cholecystolithiasis with cholecystitis, preoperative NLR and CRP levels can be used to distinguish the condition. The critical value for NLR was found to be 2.995 (95% CI, 0.9465–0.9853; *P* < 0.001) with an area under the ROC curve of 0.9659, while the critical value for CRP was 13.05 (95% CI, 0.9284–0.9830; *P* < 0.001) with an area under the ROC curve of 0.9557. Both NLR and CRP were found to have equivalent diagnostic abilities. Additionally, the study found that there were significant differences in neutrophil and lymphocyte levels in elderly patients with different severity levels, with NLR increasing as severity increased (*P* < 0.001). The study identified cut-off values for preoperative NLR that could distinguish Simple cholecystitis and Purulent cholecystitis, as well as Purulent cholecystitis and Gangrenous cholecystitis in elderly patients with cholecystolithiasis, with respective AUCs of 0.8441 (95% CI: 0.7642–0.9239; *P* < 0.001) and 0.7886(95% CI: 0.7050–0.8721, *P* < 0.001), sensitivities of 91.38% and 87.72%, and specificities of 73.33% and 63.79%.

**Conclusions:**

Preoperative NLR and CRP values can serve as indicators to detect cholecystolithiasis with cholecystitis in elderly patients. Additionally, NLR has been recognized as a potential tool to differentiate the severity of cholecystolithiasis with cholecystitis in the elderly population.

## Background

Cholecystitis is a common disease in hepatobiliary and pancreatic surgery, with gallstones obstructing the cystic duct in nearly 90% of patients due to inflammation [1, 2]. Delayed treatment can lead to complications such as gangrene, perforation, and abscess formation. Studies show that severe cholecystitis has a prevalence of 22-30%, but it can be difficult to diagnose accurately due to variable symptoms and imaging findings [3]. It is crucial to detect and treat severe cholecystitis promptly to prevent complications and avoid doctor-patient disputes in postoperative patients [4].

Gallbladder stones with cholecystitis are a prevalent biliary system disease in hepatobiliary surgery [5]. The pathogenesis of this condition involves the blockage of the cystic duct due to gallbladder stones, leading to inflammation and bile stasis. Patients typically exhibit symptoms such as Murphy’s sign or positive mass, pain, and tenderness in the right upper quadrant [6]. As the population in our country ages, the prevalence of gallstones in the elderly with cholecystitis is increasing, along with its complications, admission rate, readmission rate, and mortality rate. Due to the presence of underlying diseases in elderly patients, the gallbladder may be affected by atherosclerosis and stenosis to varying degrees [7, 8]. Additionally, some elderly patients may not be sensitive to pain, which can result in atypical symptoms or delayed medical attention. This poses a threat to their safety. Based on years of clinical confirmation, most experts in hepatobiliary and pancreatic surgery believe that cholecystectomy is a direct and effective treatment for cholecystitis [9, 10].

The neutrophil/lymphocyte ratio (NLR) has been suggested as a biomarker for systemic inflammation in recent years [11]. This type of response is characterized by an increase in neutrophils and a decrease in lymphocytes. The release of inflammation-induced arachidonic acid metabolites and platelet-activating factors, as well as cortisol-induced stress, can result in an increase in neutrophils and a decrease in lymphocytes, leading to relative lymphopenia. Therefore, NLR is considered a more accurate representation of the underlying inflammatory process [12]. C-reactive protein (CRP) plays an important role in the body’s natural immune barrier and is a clinical immunology examination. When the body experiences an inflammatory response due to infection, stress, or other reasons, CRP levels can rise rapidly. CRP is an acute-phase protein that is upregulated in response to trauma or stimulation. Research has shown that serum CRP levels are closely associated with the extent of surgical injury and serve as a sensitive indicator of tissue damage [13, 14].

The use of NLR to measure the prognosis of inflammatory and malignant diseases is increasingly supported by evidence. However, its application in elderly patients with gallstones with cholecystitis has not been reported. This study aims to evaluate the predictive value of preoperative NLR and CRP levels in elderly patients with gallstones with cholecystitis and to determine the corresponding NLR value that can distinguish between different severities of the condition. The findings of this study can provide valuable information for clinicians to predict the severity of elderly patients with gallstones with cholecystitis before surgery.

## Methods

### General information

This study was a retrospective observational study that conducted two subgroup analyses on all patients. Inpatient data for all patients were obtained from electronic medical records or admission registration medical records provided in our hospital admission records from December 2018 to November 2023. The study was approved by the Ethics Committee of the Second Affiliated Hospital of Bengbu Medical College.

We conducted a comparison of inflammatory markers between two groups: 160 elderly patients diagnosed with gallbladder stones with cholecystitis through histology (Cholecystitis group) and 60 elderly patients with normal gallbladder histology (Control group). The Cholecystitis group consisted of 70 males and 90 females with an average age of (69.14 ± 6.13), while the Control group had 29 males and 31 females with an average age of (69.20 ± 6.36).

In this study, we examined inflammatory biomarkers in elderly patients with Cholecystitis of varying severities. According to the clinical manifestations, examinations, and postoperative pathological results of cholecystitis, there were 45 cases of simple cholecystitis, 58 cases of suppurative cholecystitis, and 57 cases of gangrenous cholecystitis (According to clinical experience, pathological types of simple, purulent and gangrenous cholecystitis can only be distinguished after cholecystectomy).

### Diagnostic criteria of cholecystitis

According to the ninth edition of Surgery of People’s Health Publishing House, the diagnostic criteria of acute cholecystitis include a clinical manifestation, hematological examination, and imaging examination [15]. (1) Clinical manifestations (e.g. fever, Murphy signs or right upper abdominal mass or pain or tenderness); (2) Hematological examination (for example, elevated C-reactive protein, increased white blood cell count, and elevated serum bilirubin in about 1/2 patients); (3) Imaging examination (for example, ultrasound examination showed gallbladder enlargement, obvious edema showed bilateral sign). Diagnostic criteria for chronic cholecystitis include [16]: (1) Recurrent right upper abdominal pain that radiates to the right subscapular area and is associated with a high-fat diet; (2) There may be nausea and vomiting, the abdominal examination may have no signs or only mild tenderness in the right upper abdomen; (3) Ultrasonography showed the appearance of chronic cholecystitis.

### Inclusion and exclusion criteria

The inclusion criteria for elderly patients in the Cholecystitis group are as follows: (1) The age of the patient is greater than or equal to 60 years old; (2) Patients clinically diagnosed as cholecystolithiasis with cholecystitis; (3) All patients underwent laparoscopic cholecystectomy; and (4) All patients were examined by ultrasound (US) or abdominal CT or magnetic resonance hydrography (MRI). The exclusion criteria of patients in the Cholecystitis group were: (1) Patients under 60 years old; (2) Cholecystitis patients with other neoplastic diseases; (3) Patients with gallbladder polyps and gallbladder adenomyosis; and (4) Patients with cholelithiasis and cholangitis.

The study included a control group comprising 60 elderly patients. The selection was made randomly from the medical records of our hospital based on the histological diagnostic criteria of the normal gallbladder and the absence of other major diseases.

### Detection method and index

The admission records in our hospital were used to gather general information, disease progression, preoperative biochemical indicators, and severity classification for cholecystitis. (1) The study collected general information such as gender, age, comorbidities, and body mass index (BMI) of the participants. (2) The preoperative biochemical results were obtained by drawing 1 ml of venous blood from the patient on an empty stomach 12 h before the operation. Routine blood tests were performed by an automatic blood analyzer and the NLR was calculated. The study compared the inflammatory biomarkers NLR and CRP between the two groups. (3) In the Cholecystitis group, the course of disease refers to the period from the onset of clinical symptoms of gallbladder stones with cholecystitis to the patient’s admission to the hospital. Additionally, the histology reports were reviewed by a consultant pathologist.

### Statistical analyses

The data were collected and entered in SPSS Statistics version 26 (IBM Corp., Armonk, NY, USA). To compare the differences between the Cholecystitis group and the control group, the student’s t-test was used. In addition, a one-way analysis of variance was conducted to analyze continuous variables for intragroup comparisons of Cholecystitis of different severity. The chi-square test was used to evaluate the association between the categorical variables. *P*-value of < 0.05 was considered significant.

## Results

### Study the demographic and clinical characteristics of patients

The study included 160 elderly patients with gallbladder stones with cholecystitis with histological evidence, along with 60 patients with normal gallbladder histology. Before the operation, all patients underwent preoperative complete blood count (CBC) and CRP detection. Table 1 shows the demographic and baseline clinical characteristics of the study patients. The values of WCC, CRP, and NLR were significantly higher in elderly patients with gallbladder stones with cholecystitis compared to the control group (*P* < 0.001).

**Table 1:** Demographic and clinical characteristics of study patients

Characteristics	Cholecystitis group (n = 160)	Control group (n = 60)	*P*
Age, year (mean, SD)	69.14 ± 6.13	69.20 ± 6.36	0.952
Gender, n (%)			
Male	70(44)	29(48)	
Female	90(56)	31(52)	0.547
BMI, kg/m^2^ (mean, SD)	23.13 ± 1.61	23.18 ± 1.74	0.3664
Comorbidities, n (%)			
Hypertension, n (%)	53(32.5)	7(11.7)	0.0015
Diabetes, n (%)	69(43.1)	5(8.3)	< 0.001
TIA/stroke, n (%)	4(2.5)	1(1.6)	0.7119
Angina, n (%)	8(5.0)	0(0)	1
Arrhythmia, n (%)	7(4.3)	2(3.3)	0.7283
Heart failure, n (%)	3(1.8)	0(0)	1
Laboratory results			
Hemoglobin, g/L (mean, SD)	136.1 ± 15.48	133.6 ± 10.2	0.406
CRP, mg/L (mean, SD)	92.7 ± 65.1	4.4 ± 3.3	< 0.001
WCC, ×10^9^/L (mean, SD)	15.2 ± 8.62	8.3 ± 2.98	< 0.001
Neutrophils, ×10^9^/L (mean, SD)	6.17 ± 2.28	2.57 ± 0.81	< 0.001
Lymphocyte, ×10^9^/L (mean, SD)	1.12 ± 0.39	1.45 ± 0.15	< 0.001
NLR (mean, SD)	7.36 ± 3.73	1.78 ± 0.60	< 0.001
Total bilirubin, mmol/L (mean, SD)	17 ± 5.40	12 ± 4.39	< 0.001
GGT, U/L (mean, SD)	53 ± 12.76	61 ± 8.75	0.692
ALP, U/L (mean, SD)	92 ± 21.73	79 ± 44.93	0.274
AST, U/L (mean, SD)	28 ± 10.14	27 ± 9.08	0.619
Amylase, U/L (mean, SD)	41 ± 14.13	66 ± 37.12	< 0.001
ALT, U/L (mean, SD)	29 ± 7.29	38 ± 6.76	0.574

ALP = alkaline phosphatase; ALT = alanine transaminase; AST = aspartate aminotransferase; CRP = C-reactive protein; GGT = gamma-glutamyl transferase; SD = standard deviation; TIA = transient ischemic attack; WCC = white cell count

### Comparison of general data of elderly patients with cholecystolithiasis with cholecystitis of different severity

The study included 45 cases in the Simple cholecystitis group, consisting of 18 males and 27 females with an average age of 69.64 ± 6.22 years. The Purulent cholecystitis group included 58 cases, with 25 males and 33 females and an average age of 68.96 ± 6.22 years. The Gangrenous cholecystitis group consisted of 57 cases, with 27 males and 30 females and a mean age of 68.92 ± 6.05 years. There were no significant differences in gender, age, and BMI among elderly patients with varying severity levels (*P* > 0.05). However, there was a statistically significant difference between groups with diabetes or hypertension (*P* < 0.05). Additionally, as the severity of elderly patients with cholecystolithiasis with cholecystitis increased, the course of the disease also increased (*P* < 0.05). As shown in Table 2.

**Table 2:** Comparison of general data of elderly patients with different severity

Group	n	Gender (n)		Age, years (mean, SD)	Diabetes (n)	Hypertension (n)	The course of the disease, months (mean, SD)	BMI, kg/m^2^ (mean, SD)
		Male	Female					
Simple cholecystitis	45	18	27	69.64 ± 6.22	16	17	13.04 ± 2.95	22.66 ± 2.16
Purulent cholecystitis	58	25	33	68.96 ± 6.22	30	33	18.27 ± 3.16	22.81 ± 0.97
Gangrenous cholecystitis	57	27	30	68.92 ± 6.05	39	36	24.15 ± 3.76	23.82 ± 1.39
X^2^ or F/*P* value		0.570/0.751		0.207 /0.813	10.98/0.0041	6.878/0.0321	141.1/<0.001	1.251/0.326

### Comparison of preoperative NLR levels in elderly patients with cholecystolithiasis with cholecystitis of different severity

Table 3 shows that there were significant differences in preoperative neutrophil and lymphocyte levels and NLR among elderly patients with varying degrees of severity (*P* < 0.001). Additionally, the preoperative NLR ratio increased as the severity of the condition worsened (*P* < 0.001). A comparative analysis of the preoperative NLR was conducted among three groups of patients. The findings revealed that patients with suppurative and gangrenous cholecystitis had a higher preoperative NLR compared to patients with simple cholecystitis (*P* < 0.001). Additionally, the preoperative NLR of gangrenous cholecystitis patients was higher than that of patients with suppurative cholecystitis (*P* < 0.001).

**Table 3:** Comparison of preoperative NLR in elderly patients with different severity

Group	n	Neutrophils, ×10^9^/L (mean, SD)	Lymphocyte, ×10^9^/L (mean, SD)	NLR (mean, SD)
Simple cholecystitis	45	4.30 ± 1.57	1.10 ± 0.36	4.13 ± 1.75
Purulent cholecystitis	58	6.00 ± 1.91	1.09 ± 0.39	7.08 ± 2.33
Gangrenous cholecystitis	57	7.82 ± 1.89	1.15 ± 0.41	10.20 ± 3.85
F/*P* value		47.75/<0.001	35.62/<0.001	57.29/<0.001

### Evaluation of the differential ability of NLR in the diagnosis of cholecystitis and its comparison with CRP

The optimal cutoff value of the NLR in diagnosing Cholecystitis was 2.995 (95% CI, 0.9465–0.9853; *P* < 0.001). Sensitivity at the cutpoint was 100%, specificity was 85.63%, and the AUC was 0.9659.

**Fig. 1:**
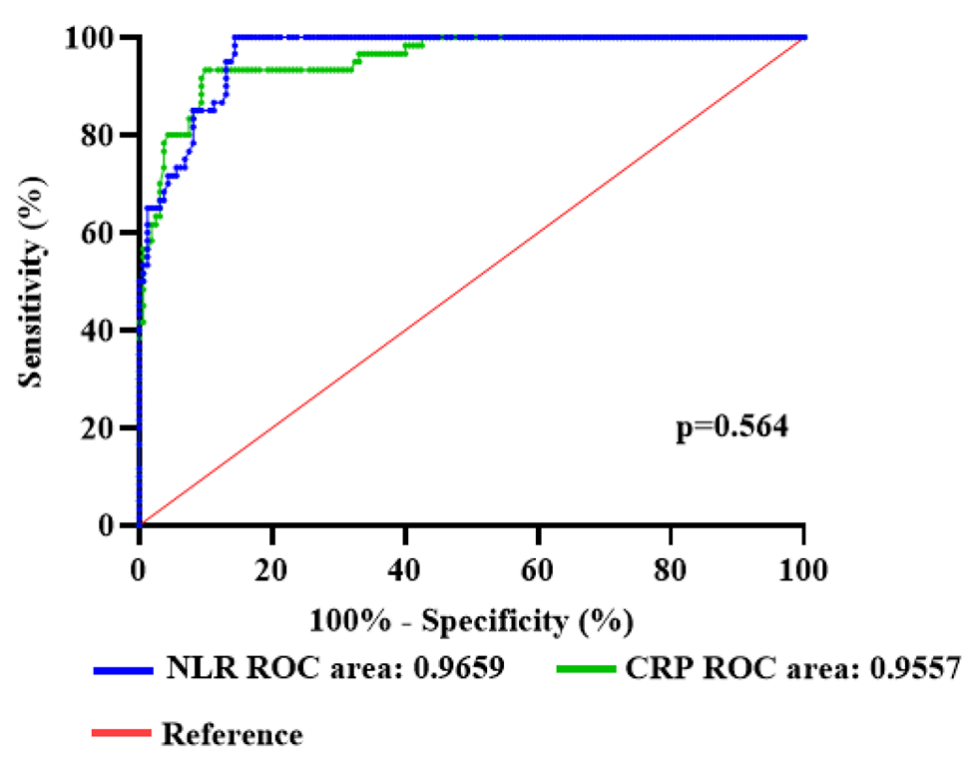
Application of ROC curve of NLR and CRP in the diagnosis of elderly patients with cholecystolithiasis with cholecystitis

The optimal cutoff value of the CRP in diagnosing Cholecystitis was 13.05 (95% CI, 0.9284–0.9830; *P* < 0.001). Sensitivity at the cutpoint was 93.33%, specificity was 90.00%, and the AUC was 0.9557. In these patients, NLR showed similar discrimination to CRP. (*P* = 0.564). As shown in Fig. 1.

### Prediction of the severity of senile cholecystolithiasis with cholecystitis by preoperative NLR

The study found that a preoperative NLR value of 5.061 was the best cut-off point to differentiate patients with Simple cholecystitis and Purulent cholecystitis severity. The AUC was 0.8441 (95% CI: 0.7642–0.9239; *P* < 0.001), indicating good accuracy. The sensitivity and specificity were determined to be 91.38% and 73.33%, respectively.

**Fig. 2:**
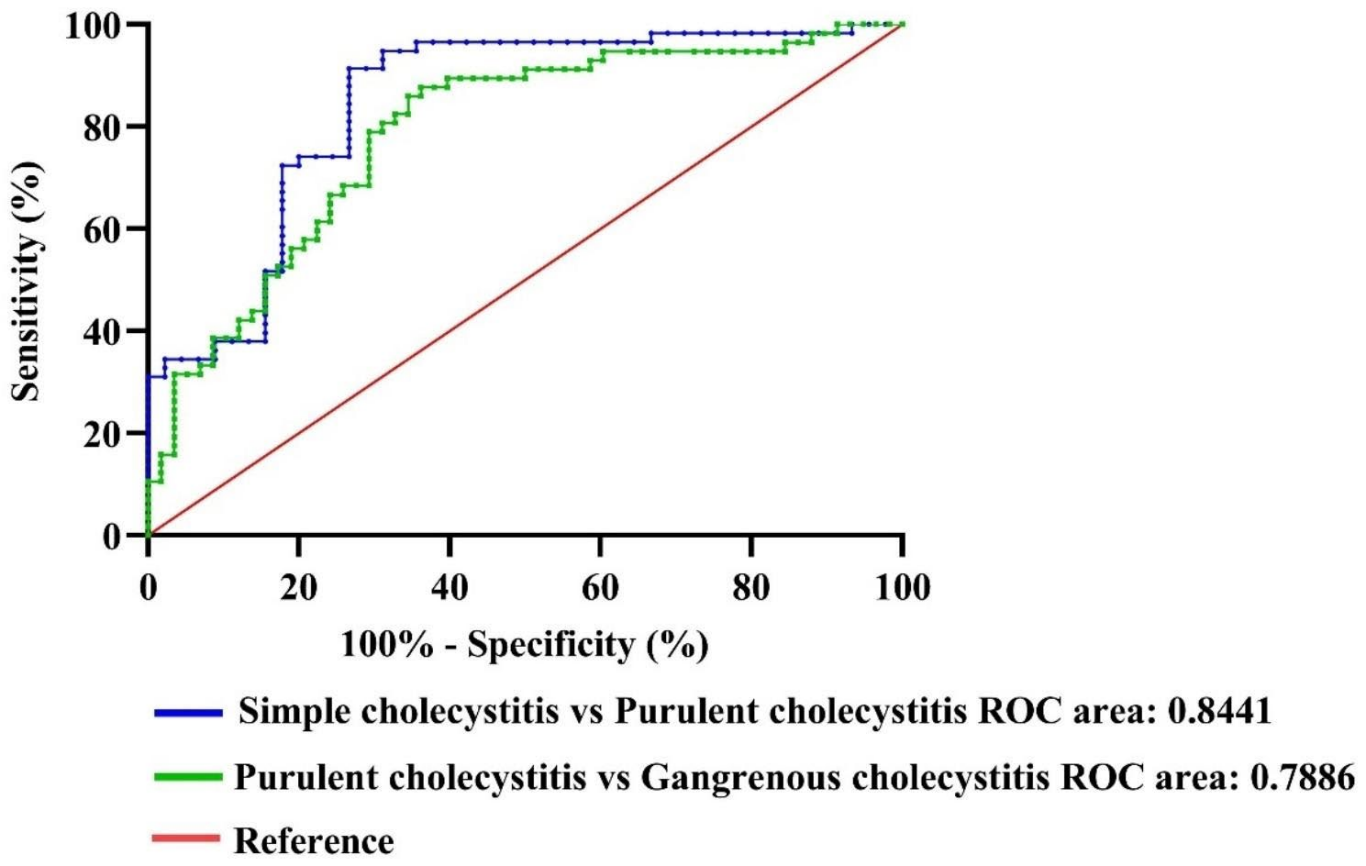
Preoperative NLR ROC curve of simple cholecystitis and purulent cholecystitis, purulent cholecystitis, and gangrenous cholecystitis severity in elderly patients with cholecystolithiasis with cholecystitis

To differentiate patients with Purulent cholecystitis and Gangrenous cholecystitis severity, a preoperative NLR cut-off value of 7.248 was found to be optimal with an AUC of 0.7886 (95% CI: 0.7050–0.8721, *P* < 0.001). The sensitivity and specificity were determined to be 87.72% and 63.79%, respectively, as shown in Fig. 2.

## Discussion

Gallbladder stones are a prevalent global health issue and are commonly encountered in clinical practice within my country. The etiology of gallstones is multifactorial and can be attributed to factors such as obesity and high-fat diets. Cholecystitis, characterized by gallbladder inflammation, is typically caused by cystic duct obstruction from gallstones and can have a prolonged course with a high risk of recurrence [17, 18]. Research indicates that approximately 95% of cholecystitis cases are associated with gallstones [19]. Delayed intervention can result in chronic cystic bile duct obstruction, leading to gallbladder lumen dilation and wall enlargement due to submucosal edema, which can exacerbate the physical and psychological burden on patients and complicate treatment. Therefore, timely intervention is crucial to prevent further complications [20, 21].

Elderly patients have a higher incidence of biliary tract diseases, with inflammation worsening with age. Gallbladder stones accompanied by severe cholecystitis can lead to serious complications such as empyema, gallbladder gangrene, and gallbladder perforation. In addition, the occurrence of other complications such as high blood pressure, heart disease, diabetes, chronic lung disease, and abnormal renal function also increases [22]. However, due to the sluggish response of the elderly body, typical symptoms and signs of the disease may not be present during diagnosis, making clinical diagnosis and treatment more difficult. Proper identification and management are crucial in avoiding multiple comorbidities, defining the boundaries of cholecystectomy and conservative treatment, and improving disease prognosis and care [23]. In patients with severe cholecystitis, there is a significantly increased risk of bile duct and hepatic duct injury during surgery. The study revealed that more than half of elderly patients with gallbladder stones with cholecystitis had underlying diseases, particularly diabetes, and hypertension [24].

Imaging examinations such as ultrasound (US), CT, MRI, and magnetic resonance cholangiopancreatography (MRCP) are currently used to detect severe cholecystitis, but they are not always sensitive enough [25]. Recent studies have shown that the presence of systemic inflammatory responses is linked to clinical signs and poor prognosis in patients with different types of inflammatory diseases and malignancies [26]. Various prognostic scoring criteria based on inflammation have been proposed, such as the modified Glasgow prognostic score (mGPS), peripheral blood platelet-to-lymphocyte ratio (PLR), NLR, prognostic nutritional index, and CRP [27]. Studies have shown that these scores are strongly associated with the outcome or disease severity in patients with appendicitis, sepsis, heart failure, and sepsis [28, 30]. One important limitation of these studies is the lack of exclusion of patients with diseases that may impact blood values. Serum levels can be influenced by various conditions, including malignancy or inflammatory disease, ongoing chemotherapy or radiation therapy, pregnancy, known hematological disorders, the presence of multiple comorbidities, and the use of medications [31].

NLR has gained significant attention in clinical practice due to its use of standard whole blood cell technology, easy calculation, and low cost. Neutrophils and lymphocytes play crucial roles in the inflammatory response, immune response, and blood coagulation reaction [32]. Neutrophils make up 50-70% of all leukocytes in human circulation and are believed to promote cancer cell proliferation, angiogenesis, and metastasis by producing pro-angiogenic chemokines and vascular endothelial growth factors. On the other hand, lymphocytes in peripheral blood are thought to exert tumor-suppressive properties and cause synergistic cytotoxicity. Patients with advanced inflammatory diseases and malignant tumors often exhibit elevated levels of NLR as a typical manifestation of systemic inflammatory response. This is due to the presence of pro-inflammatory cytokines in their blood, which can cause changes in the tissue microenvironment [33]. These changes can be used to stratify cancer and are often associated with tumor size, stage, metastatic potential, and lymphatic infiltration. Neutropenia and lymphopenia are frequently seen in critically ill patients with sepsis and have been linked to higher mortality rates [34]. Therefore, healthcare professionals continuously monitor the absolute numbers of neutrophils and lymphocytes in these patients, as they can serve as independent prognostic tools. However, in contrast to this, the NLR is a readily available measurement that combines the quantification of neutrophils and lymphocytes into a ratio that indicates the innate and adaptive immune system’s response to inflammation, infection, and tissue damage [35].

It is believed that measuring regulatory response may prove to be a useful biomarker for predicting severity in elderly patients with cholecystolithiasis with cholecystitis. The findings of this study indicate that preoperative NLR can serve as a reliable predictor of cholecystolithiasis with cholecystitis in elderly patients. The study determined that the optimal cut-off value of NLR for diagnosing cholecystitis was 2.995 (95% CI: 0.9465–0.9853; *P* < 0.001), with an AUC of 0.9659. Similar results were observed in other studies, where the optimal cut-off value of preoperative NLR for elderly patients with cholecystolithiasis with cholecystitis was 2.73 (*P* < 0.05), and the AUC was 0.778. The application of surgery inevitably causes trauma to the patient’s body, resulting in the body’s inflammatory stress response. The level of inflammatory factors reflects the extent of trauma caused by the surgery. CRP is an acute-phase protein synthesized in the liver and released into the blood. It is released in response to external trauma and tissue damage. Excessive release of inflammatory factors hinders the postoperative recovery of body tissues [36]. This study also found that NLR had comparable predictive power to CRP. The optimal cut-off value for CRP diagnosis was 13.05 (95% CI: 0.9284–0.9830; *P* < 0.001), with an AUC of 0.9557. In these patients, NLR showed similar discrimination as CRP (*P* = 0.564). Various studies have indicated that the cut-off values of NLR and CRP vary depending on the type and severity of the disease.

Gallbladder gangrene is a severe complication of cholecystitis that occurs due to inadequate blood supply and ischemia, resulting in necrosis and potential perforation of the gallbladder wall. Accurately predicting the condition of the gallbladder before surgery is challenging but crucial for evaluating the patient’s status [37]. This study found that a preoperative NLR value of 5.061 is the optimal threshold for distinguishing between simple cholecystitis and suppurative cholecystitis, with an AUC of 0.8441 (95% CI: 0.7642–0.9239; *P* < 0.001), indicating good accuracy. The sensitivity and specificity were 91.38% and 73.33%, respectively. To differentiate between suppurative cholecystitis and gangrenous cholecystitis, a preoperative NLR cutoff of 7.248 was identified as optimal, with an AUC of 0.7886 (95% CI: 0.7050–0.8721, *P* < 0.001). The sensitivity and specificity were 87.72% and 63.79%, respectively. These findings align with previous research, which also noted higher preoperative NLR values in patients with purulent cholecystitis and gangrenous cholecystitis compared to those with simple cholecystitis (*P* < 0.05). Additionally, the preoperative NLR was higher in patients with gangrenous cholecystitis compared to those with suppurative cholecystitis (*P* < 0.05). The AUC values for NLR in patients with gangrenous cholecystitis and suppurative cholecystitis were 0.920 and 0.676, respectively, indicating high accuracy [38]. As a result, preoperative NLR is a valuable tool in accurately determining the severity and prognosis of elderly patients with cholecystolithiasis with cholecystitis.

## Conclusions

This study suggests that preoperative NLR and CRP values can serve as a simple method for identifying the severity of cholecystitis in elderly patients with cholecystolithiasis. Additionally, they can also be used as surrogate indicators for forecasting. The study found that a preoperative NLR cutoff value of 2.995 and a CRP cutoff value of 13.05 can distinguish the occurrence of the disease in elderly patients with cholecystolithiasis with cholecystitis. Furthermore, a preoperative NLR cutoff value of 5.061 can distinguish between simple cholecystitis and purulent cholecystitis in elderly patients with cholecystolithiasis, while a critical value of preoperative NLR at 7.248 can distinguish between suppurative cholecystitis and gangrenous cholecystitis in elderly patients with cholecystolithiasis.

## Data Availability

The datasets used and analyzed in this study are available from the corresponding author upon reasonable request.

## Abbreviations

NLR: Neutrophil/lymphocyte ratio
CRP: C-reactive protein
BMI: Body mass index
ALP: Alkaline phosphatase
ALT: Alanine transaminase
AST: Aspartate aminotransferase
CRP: C-reactive protein
GGT: Gamma-glutamyl transferase
SD: Standard deviation
TIA: Transient ischemic attack
WCC: White cell count
US: Ultrasound
MRCP: Magnetic resonance cholangiopancreatography
mGPS: Modified Glasgow prognostic score
PLR: Peripheral blood platelet-to-lymphocyte ratio
